# Novel Real-Time OEP Phase Angle Feedback System for Dysfunctional Breathing Pattern Training—An Acute Intervention Study

**DOI:** 10.3390/s21113714

**Published:** 2021-05-26

**Authors:** Carol M. E. Smyth, Samantha L. Winter, John W. Dickinson

**Affiliations:** 1 School of Sport & Exercise Sciences, University of Kent, Canterbury CT2 7NB, UK; c.m.e.smyth@kent.ac.uk; 2 School of Sport, Exercise and Health Sciences, Loughborough University, Loughborough LE11 3TU, UK; s.l.winter@lboro.ac.uk

**Keywords:** optoelectronic plethysmography, dysfunctional breathing, phase angle, exercise, intervention

## Abstract

Dysfunctional breathing patterns (DBP) can have an impact on an individual’s quality of life and/or exercise performance. Breathing retraining is considered to be the first line of treatment to correct breathing pattern, for example, reducing ribcage versus abdominal movement asynchrony. Optoelectronic plethysmography (OEP) is a non-invasive 3D motion capture technique that measures the movement of the chest wall. The purpose of this study was to investigate if the use of a newly developed real-time OEP phase angle and volume feedback system, as an acute breathing retraining intervention, could result in a greater reduction of phase angle values (i.e., an improvement in movement synchrony) when compared to real-time OEP volume feedback alone. Eighteen individuals with a DBP performed an incremental cycle test with OEP measuring chest wall movement. Participants were randomly assigned to either the control group, which included the volume-based OEP feedback or to the experimental group, which included both the volume-based and phase angle OEP feedback. Participants then repeated the same cycle test using the real-time OEP feedback. The phase angle between the ribcage versus abdomen (RcAbPhase), between the pulmonary ribcage and the combined abdominal ribcage and abdomen (RCpAbPhase), and between the abdomen and the shoulders (AbSPhase) were calculated during both cycle tests. Significant increases in RcAbPhase (pre: −2.89°, post: −1.39°, *p* < 0.01), RCpAbPhase (pre: −2.00°, post: −0.50°, *p* < 0.01), and AbSPhase (pre: −2.60°, post: −0.72°, *p* < 0.01) were found post-intervention in the experimental group. This indicates that the experimental group demonstrated improved synchrony in their breathing pattern and therefore, reverting towards a healthy breathing pattern. This study shows for the first time that dysfunctional breathing patterns can be acutely improved with real-time OEP phase angle feedback and provides interesting insight into the feasibility of using this novel feedback system for breathing pattern retraining in individuals with DBP.

## 1. Introduction

Dysfunctional breathing is defined as chronic alterations in breathing pattern which may present as hyperventilation, an asynchronous breathing pattern, and/or a thoracic dominant breathing pattern, such that the contribution of the upper chest compartment to the total breath volume is greater compared to healthy individuals [1]. Dysfunctional breathing patterns (DBP) can negatively impact an individual’s quality of life, with individuals experiencing breathing problems impairing daily life activities and/or exercise performance [2,3]. Dysfunctional breathing can often occur alongside other respiratory diseases/disorders further exacerbating the disease/disorder, for example, asthmatics with dysfunctional breathing experience an increase in self-reported symptoms [4], medication use, and a reduction in asthma control [5,6].

Breathing pattern retraining is considered to be the first line of treatment of dysfunctional breathing [7]. Breathing pattern retraining aims to progressively alter and retrain an individual’s breathing pattern, in order to restore and maintain a healthy, normal breathing pattern through the use of breathing exercises [8]. Breathing exercises can focus on a number of different factors including reducing respiratory rate and tidal volume [7], altering breathing pattern, improving respiratory muscle strength and/or endurance, improving posture, and/or increasing the range of motion of the thorax [9].

It has previously been established within the literature that a normal breathing pattern can be described as the pulmonary ribcage, abdominal ribcage, and the abdomen moving in synchronization [10]. Achieving such a breathing pattern through breathing retraining has been investigated with techniques, such as the Buteyko breathing [11], diaphragmatic breathing [3], the Papworth method [12], and yoga breathing [13]. However, there has been no consensus on the use of breathing exercises within clinical practice [8,14]. Additionally, flow-incentive and volume-incentive spirometry feedback have been investigated as potential breathing retraining aids, with volume-incentive spirometry promoting deeper and slower breathing in healthy individuals [15] and in patients post-gastroplasty [16]. This indicates that different forms of feedback mechanisms used in breathing training can have a different impact on breathing pattern retraining.

The primary outcomes for many breathing pattern retraining studies are self-reported questionnaires such as the asthma quality of life questionnaire [17], the Nijmegen questionnaire [3], or the self-evaluation of breathing questionnaire [5]. Bruton and colleagues [17] demonstrated that quality of life scores improved and reduced adverse events in asthmatics receiving breathing pattern training versus standard care. Similarly, a five-year follow-up breathing retraining study for individuals with dysfunctional breathing demonstrated significant improvements in quality of life scores, reduced symptoms, and reduced emergency room visits [3]. Additionally, the manual assessment of respiratory motion has previously indicated reduced asynchrony between the pulmonary ribcage and the combined abdominal ribcage and abdomen, post breathing retraining in asthmatics with dysfunctional breathing [5]. From these results, breathing pattern retraining can reduce the asynchrony associated with dysfunctional breathing which leads to a subsequent reduction in symptoms and improved quality of life scores.

Currently, there are a number of different non-invasive systems that can be used to measure breathing patterns, including optoelectronic plethysmography (OEP) [18], structured light plethysmography (SLP) [19], and respiratory inductive plethysmography (RIP) [20]. These systems allow for the objective measurement of thoracic compartment asynchronies in a wide range of pathological and non-pathological population groups [16,19,21]. Wearable technologies have also shown some promise in this area, for example, a smart textile consisting of 12 fibre Bragg grating sensors have demonstrated good agreement with OEP when measuring breathing patterns at rest [22]. More recently, phase-comparison monopulse radar has been used to successfully detect normal, fast, and slow breathing patterns non-invasively in healthy participants [23]. OEP has the advantage over these similar measurement systems as it can be used and has been previously validated during exercise [21,24], making it more suitable for certain population groups prone to dysfunctional breathing, such as athletes with dysfunctional breathing.

Optoelectronic plethysmography (OEP) is a non-invasive 3D motion capture technique often used to measure the movement of the chest wall. This 3D motion capture technique utilizes infrared cameras to track and record the 3D coordinates of retro-reflective markers placed on the torso. This allows breathing patterns to be measured objectively and non-invasively. OEP has previously been used to assess different types of breathing interventions including breath/air stacking [25,26], incentive spirometry [15], loaded inspiratory breathing [27], and rehabilitation [28]. OEP has also been used to investigate the impact of different types of breathing exercises including diaphragmatic breathing, inspiratory sighs, sustained maximal inspiration, and intercostal breathing in healthy individuals [29]. These breathing exercises were shown to alter OEP-derived breathing parameters, such as respiratory rate, tidal volume, thoracic compartment contribution, and phase angles, when compared to tidal breathing [29].

**Figure 1:**
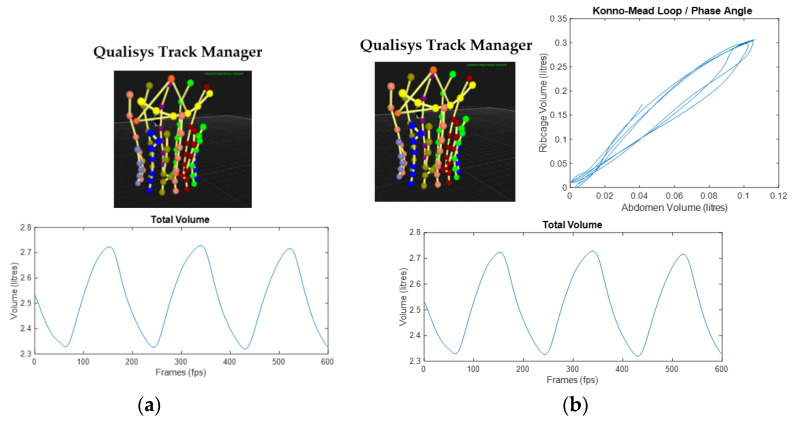
OEP real-time feedback system displayed for: (**a**) the control group with Qualisys Track Manager attaching the AIM model to the 90 markers and the real-time total volume trace plot streamed via MATLAB; (**b**) the experimental group with the additional real-time Konno–Mead breath loop for the phase angle between the ribcage and abdomen streamed via MATLAB.

Phase angle is a measure of the temporal movement of one torso compartment in relation to another during each breath (Equation (1)). A phase angle of zero represents perfect synchrony between two compartments such that they move together during inhale and exhale. A phase angle of ±90° would represent perfect asynchrony. Phase angles can also be visually represented as Konno–Mead [30] loops (Figure 1b). OEP-derived phase angles have been shown to significantly change with breathing exercises, for example, the phase angle between the ribcage and abdomen, and between the upper and lower ribcage [29]. Similarly, RIP-derived phase angle between the ribcage and abdomen has been shown to change between breathing training methods such as diaphragmatic breathing, flow-oriented spirometry, and volume-oriented spirometry in obese patients post gastroplasty [16]. In addition, OEP-derived phase angles have previously been used to distinguish between individuals with and without a DBPs at rest and during exercise [21].

Overall, the evidence suggests that breathing pattern retraining can be used to alter DBPs [3]. From previous literature, volume-based feedback systems are promoted over alternatives such as flow-based feedback for breathing training, as it results in more controlled breaths and reduced breathing patten asynchrony [15,16]. Objective breathing pattern measurement systems, including OEP, have not been used to assess the influence of breathing retraining on the breathing pattern of individuals with dysfunctional breathing, nor has OEP been used to provide real-time feedback on breathing pattern to any population group.

This study for the first time presents a real-time OEP feedback system. This real-time OEP feedback system has been developed with the ability to display both volume and phase angle feedback in real-time. To our knowledge, phase angle feedback has never been used in real-time as part of breathing retraining intervention. It was hypothesized that real-time OEP phase angle feedback between the ribcage and abdomen (RcAbPhase) along with real-time volume feedback, as part of an acute breathing retraining intervention, will result in a greater reduction in the RcAbPhase value (i.e., closer to zero and perfect synchrony) at rest and during exercise when compared to real-time OEP volume-based feedback alone.

## 2. Materials and Methods

### 2.1. Participants

Eighteen participants with a suspected DBP gave written informed consent to participate in this study which was approved by the School of Sport and Exercise Sciences Research Ethics Advisory Group at the University of Kent, UK (Prop 92_2018_19). Inclusion criteria included a negative eucapnic hyperpnoea test [31,32] and reporting respiratory symptoms at rest and/or exercise. Participants were asked to report on the following symptoms: coughing, wheezing breathing in and/or out, chest tightness breathing in and/or out, dyspnoea, and excess mucus production during or post-exercise. Participants were randomly assigned to either the control group (n = 9; age 28.6 ± 10.7 years; height 1.7 ± 0.1 m; body mass 63.6 ± 11.3 kg), which included the volume-based OEP feedback system, or to the experimental group (n = 9, age 25.0 ± 8.2 years; height 1.7 ± 0.1 m; body mass 70.2 ± 13.6 kg), which included both the volume-based and phase angle OEP feedback system. Participants performed a sub-maximal exercise challenge, both groups then received the same breathing retraining instructions, and repeated the same exercise challenge with the corresponding real-time OEP feedback.

### 2.2. Equipment

The OEP system consisted of 11 cameras (Qualisys, Goteborg, Sweden) sampling at 100 Hz and were positioned around a cycle ergometer (Lode-Corival). Calibration of the OEP system was performed prior to each participant and was only accepted if the average residuals for each camera were below 1.0 mm. Ninety markers were placed on the torso in a grid-like pattern. This OEP marker set has previously been validated against breath-by-breath analysis at rest and during exercise [18,33]. This marker set allows for the division of the torso into the pulmonary ribcage (RCp), the abdominal ribcage (RCa), and the abdomen (AB).

### 2.3. Protocol

Tidal breathing was measured at rest and during a series of sub-maximal exercise intensities on a cycle ergometer. The participants were positioned upright on the cycle ergometer with arms extended to the side and rested on stands for optimal marker visibility. The cycle test began at 50 W and increased by 30 W every minute. Breathing pattern data was recorded using the OEP for approximately 30–60 s at rest, during exercise (low, moderate, and high intensity), and recovery immediately post exercise. The exercise intensities were defined using rate of perceived exertion values of 11, 13/14, and 17/18 representing low, moderate, and high intensity exercise, respectively [34]. Once recovered from the cycle test, all participants took part in an acute breathing retraining intervention. Post-intervention, participants repeated the cycle test with the novel real-time OEP feedback system. The real-time visual feedback was displayed throughout the cycle test. Breathing pattern data was recorded again using the OEP system for approximately 30–60 s at rest, during high intensity exercise, and recovery post-exercise.

### 2.4. Breathing Retraining Intervention

Both the control and experimental group were given the same set of breathing retraining instructions which included focusing on using the diaphragm and intercostal muscles to initiate the movement of the breath, move the lower ribcage laterally, avoid initiating the breath from the shoulders and abdomen, move the ribcage and abdomen together, and aim for smooth inhalation using the real-time OEP feedback as an aid. The control group used a real-time OEP plot of the total volume trace (Figure 1a). The experimental group used an additional real-time OEP plot of the Konno–Mead breath loop associated with the ribcage and abdomen phase angle (Figure 1b), with the added instruction to follow a straight 45-degree line within the Konno–Mead plot.

The real-time OEP feedback system was developed using QTM Connect for MATLAB (Qualisys, Goteborg, Sweden) and a custom-built MATLAB (v2019a, Mathworks Inc., Natick, MA, USA) script. This allowed for the streaming of the OEP data to be viewed by the participants in real-time. Participants were given time to practice implementing the feedback and using the real-time feedback system for as long as required, typically approximately 30 min. Once comfortable with the instructions and feedback system, the participants repeated the cycle test while viewing the real-time OEP feedback.

### 2.5. Data Analysis

**Figure 2:**
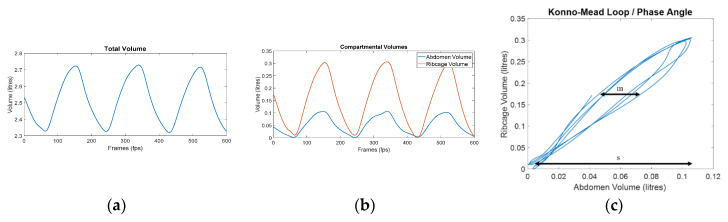
Example of OEP signal processing for phase angle calculation: (**a**) total volume trace calculated from the 90 markers using the prism-based method [33]; (**b**) division of the total volume into the ribcage and abdomen compartmental volumes; (**c**) Konno–Mead loop representing the phase angle between the ribcage and abdomen (RcAbPhase). m is the width of the loop at 50% of the ribcage displacement, s is the range of abdomen displacement, and phase angle is calculated as arcsin (m/s).

**Figure 3:**
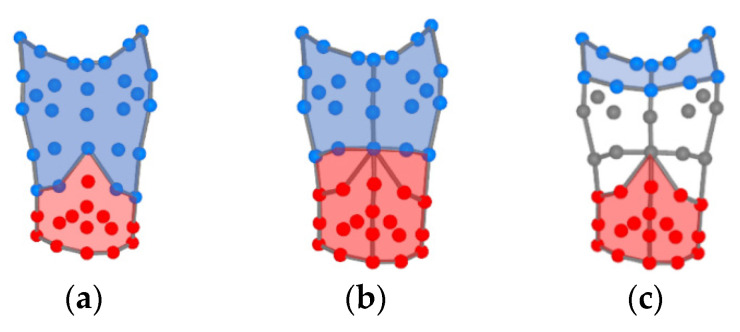
Division of the OEP marker set (anterior view) into: (**a**) the ribcage (blue) and abdomen (red) for the phase angle RcAbPhase; (**b**) the pulmonary ribcage (blue) and the combined abdominal ribcage and abdomen (red) for the phase angle RCpAbPhase; (**c**) the shoulders (blue) and the abdomen (red) for the phase angle AbSPhase.

The OEP trials were gap filled for up to 10 frames using Qualisys Track Manager v2019.3. Total volume and compartmental volume were calculated using the prism-based method [33]. Respiratory rate (RR) was calculated from the OEP volume trace. The phase angle between two compartments was calculated using the following equation:

Phase angle = arcsin (m/s)(1)

where s is the range of the first compartment displacement and m is the width of the loop at 50% of the second compartment displacement [19,21]. The phase angle calculation is summarized in Figure 2 and can be performed with any two torso compartments. This study calculated the phase angle between the ribcage and the abdomen compartments (RcAbPhase), between the pulmonary ribcage and the combined abdominal ribcage and abdomen compartments (RCpAbPhase), and between the abdomen and the shoulder compartments (AbSPhase). Figure 3 illustrates the division of the associated compartments for each phase angle calculated in this study. A mixed model repeated measures ANOVA was used to determine if there was an interaction effect between the intervention time points, i.e., pre- and post-breathing retraining and the group. Follow-up paired sample t-tests were performed to determine if there were significant differences within each group pre- and post- the acute breathing retraining intervention.

## 3. Results

**Figure 4:**
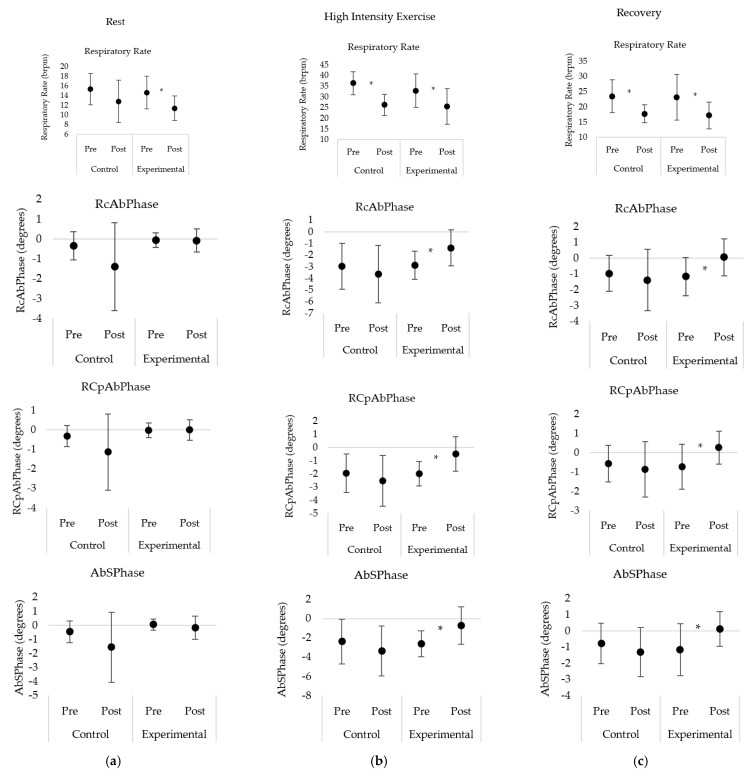
Comparison of breathing parameters between the control and experimental group at: (**a**) rest; (**b**) high intensity exercise; and (**c**) recovery post exercise pre- and post- acute breathing retraining intervention. * represents significant post hoc contrasts between pre- and post-intervention conditions within a given group with *p* < 0.05.

Respiratory rate (RR) displayed no significant interaction effect at rest, during exercise, and recovery post-exercise. During rest, both groups demonstrated a decrease in RR from pre- to post-intervention, with this decrease being significant for the experimental only. During both high intensity exercise and recovery, both groups displayed a significant decrease in RR from pre- to post-intervention (Table 1, Figure 4).

During the resting condition, both groups displayed no significant interaction effect and no significant differences between each of the phase angles within each group (Table 1, Figure 4).

During high intensity exercise and recovery post-exercise, there was a significant interaction effect for each of the phase angles measured (RcAbPhase, RCpAbPhase, and AbSPhase). More specifically, the experimental group displayed significant increases in the RcAbPhase phase angle value post-intervention (Table 1, Figure 4) during high intensity exercise (*p* = 0.002) and recovery (*p* < 0.001). This indicates a reduction in asynchrony between the ribcage and abdomen post-intervention. During high intensity exercise, the abdomen initiated the movement of the breath for both pre- and post-conditions; while for the recovery condition, the abdomen initiated the breath pre-intervention, but post-intervention the ribcage initiated the breath. In contrast, the control group displayed non-significant differences in phase angle values from pre to post-intervention.

Similarly, the experimental group displayed significant increases in the RCpAbPhase values during high intensity exercise (*p* = 0.002) and recovery (*p* = 0.001) post-intervention (Table 1, Figure 4). This demonstrates that the asynchrony between the pulmonary ribcage and the combined abdominal ribcage and abdomen reduced post-intervention with the combined abdominal ribcage and abdomen initiating the breath during high intensity exercise. The control group demonstrated non-significant differences in phase angle values for RCpAbPhase during high intensity exercise and recovery.

Finally, for the phase angle AbSPhase, the experimental group displayed a significant increase in phase angle values post-intervention (Table 1, Figure 4) during high intensity exercise (*p* = 0.005) and recovery (*p* = 0.001). This demonstrates a significant reduction in the asynchrony between the abdomen and shoulder compartments. During high intensity exercise, the abdomen initiated the movement of the breath for both pre- and post-intervention conditions. While during recovery, the abdomen initiated the breath pre-intervention and the shoulders initiated the breath post-intervention. The control group displayed non-significant differences in AbSPhase values during rest, high intensity exercise and recovery.

## 4. Discussion

For the first time, this study demonstrates that dysfunctional breathing patterns can be acutely improved with breathing retraining using a novel real-time OEP phase angle feedback. This novel application of OEP was used as part of a breathing pattern acute intervention for individuals with a DBP at rest, during exercise, and recovery immediately post-exercise. The findings demonstrate that the real-time OEP phase angle feedback with the volume feedback can significantly improve the phase angle values between the ribcage and abdomen (RcAbPhase), the pulmonary ribcage and combined abdominal ribcage and abdomen (RCpAbPhase), and the abdomen and shoulders (AbSPhase) during high intensity and recovery post-exercise. This improvement brought the phase angle values closer to zero indicating a reduction in compartment asynchrony, and therefore, a reversion towards a normal breathing pattern.

The respiratory rate (RR) decreased significantly post-intervention for both groups during high intensity exercise and recovery post-exercise (Table 1). This indicates that both forms of real-time feedback aided the control of respiratory rate in individuals with a DBP. DBPs may present in individuals as chronic changes in breathing pattern, and hyperventilation is commonly associated with this [1]. Reducing respiratory rate is a common goal of breathing retraining [7] as it improves control and reduces symptoms, such as dizziness [35]. Previously, diaphragmatic, inspiratory sighs, sustained maximal inspiration, and intercostal breathing have all been shown to significantly reduce OEP-derived respiratory rate in healthy individuals [29]. Similarly, volume-based spirometry reduced respiratory rate obtained from RIP in obese patients when compared to flow-incentive spirometry [16]. Using the Nijmegen Questionnaire, breathing exercises have been shown to reduce symptoms of hyperventilation and improve breathing control in asthmatics [5] and dysfunctional breathing [3]. This study is comparable to the previous evidence within the literature and indicates that the OEP feedback system, both phase angle and volume, can aid in reducing respiratory rate in individuals with DBPs during rest, exercise, and recovery post-exercise.

Previous research has demonstrated from OEP-derived phase angles that the asynchrony between thoracic compartments can increases with exercise in individuals with DBPs [21]. This study follows a similar trend with both the control and experimental group demonstrating an increase in compartment asynchrony from rest to high intensity exercise during both exercise tests (Table 1). RcAbPhase is a measure of the thoracoabdominal asynchrony which is commonly reported within the literature and may be classified as a sub-type of dysfunctional breathing [1]. At rest, there were no significant differences between pre- and post-intervention conditions for both groups, and no interaction effect for RcAbPhase (Table 1). In the experimental group, RcAbPhase displayed significant increase in phase angle with a value closer to zero, i.e., perfect asynchrony post-intervention during high intensity exercise and recovery post-exercise (Figure 4). In contrast, RcAbPhase did not differ significantly during high intensity exercise and recovery for the control group (Figure 4). It has previously been reported that breathing exercises including diaphragmatic and intercostal breathing significantly increases OEP-derived RcAbPhase values in healthy individuals indicating more asynchrony [29]. Similarly, in obese patients, flow and volume incentive spirometry increases the asynchrony between the ribcage and abdomen in relation to RIP-derived RcAbPhase. The results of this study support the hypothesis that the real-time OEP phase angle feedback would result in reduced RcAbPhase values and therefore, improved ribcage and abdomen synchrony in individuals with a DBP. This demonstrates that individuals with a DBP were able to utilize this novel feedback system to alter and improve the synchrony between the ribcage and abdomen, therefore, reverting towards a healthy breathing pattern.

Similar to RcAbPhase, the phase angle between the pulmonary ribcage and the combined abdominal ribcage and abdomen (RCpAbPhase) demonstrated phase angle values significantly closer to zero in the experimental group post-intervention during high intensity exercise and recovery post-exercise (Figure 4). This indicates that participants were able to reduce the asynchrony between these compartments using the real-time OEP phase angle feedback system. In contrast, the control group displayed non-significant differences for RCpAbPhase during high intensity exercise and recovery. Due to the similarities between RcAbPhase and RCpAbPhase, it would be expected that they would have a comparable response to breathing retraining. More specifically, if one improved in terms of compartment asynchrony, the other would also improve, as seen in this study. Previously, OEP-derived RCpAbPhase has been reported to increase during flow and volume incentive spirometry, although not significantly, in healthy adults [16]. Other systems such as structured light plethysmography and respiratory inductive plethysmography can measure RcAbPhase and RCpAbPhase, however, OEP is the only system that can be accurately used during exercise [24]. Currently, OEP is also the only system with the ability to display real-time phase angle feedback.

Similar to the other phase angles in this study, AbSPhase demonstrated phase angle values significantly closer to zero in the experimental group post-intervention during high intensity exercise and recovery (Figure 4). AbSPhase represents the phase angle between the abdomen compartment and the shoulders. This novel phase angle was developed to quantify thoracic dominant breathing pattern, also referred to as apical breathing and currently can only be measured using OEP. Prior to this study, similar shoulder related phase angles have shown significant differences between individuals with and without a DBP [21]. Thoracic dominant DBPs involve an increase in the vertical motion of the ribcage with minimal abdomen movement [1]. This motion can increase the activation of muscles, such as the upper trapezius, which can increase the elevation of the shoulders [35]. This common characteristic of DBP may cause postural issues and/or shoulder pain [35]. Individuals with a thoracic dominant DBP also score highly on the Nijmegen questionnaire and may be exacerbated with increased ventilation demand, such as during exercise [1]. AbSPhase increased from rest to high intensity exercise during both pre- and post-intervention cycle tests. However, during high intensity exercise AbSPhase was significantly closer to zero post-intervention in the experimental group only (Figure 4). Previous research has used breathing exercises such as diaphragmatic breathing to successfully improve thoracic dominant breathing in patients with chronic obstructive pulmonary disease [36]. The alterations in AbSPhase values during high intensity exercise and recovery post-exercise in this study indicates that the experimental group successfully used the real-time OEP phase angle feedback to improve the synchrony between the shoulders and abdomen and therefore, reduce thoracic dominant DBP.

The results of this study indicate that individuals with DBPs can manipulate their breathing pattern with the use of real-time OEP phase angle visual feedback, to revert towards a more synchronous, normal breathing pattern. Not only did the phase angle values move closer to zero, but in some cases, the order in which the compartments moved changed in the experimental group. During recovery, the abdomen became the breath initiating compartment across each of the phase angles in the experimental group only (Table 1). This may indicate that using the phase angle feedback aided the experimental group to alter their breathing pattern with more abdominal movement and less thoracic excursion and therefore, reducing thoracic dominant DBP.

However, one of the main limitations of OEP and the real-time feedback system is that in order to successfully track each marker optimally, participants often have to extend their arms to the side. If the arms are not extended, the markers on the side of the torso may be obscured. This is an unnatural position, particularly during exercise, and has the potential to alter activation of inspiratory muscles and, thus, potentially alter breathing pattern. To minimize this, participants rested their arms on stands rather than actively holding them out.

## 5. Conclusions

In conclusion, this study demonstrates that the use of this newly developed real-time OEP phase angle feedback system as part of an acute breathing retraining intervention for individuals with DBP results in altered phase angle values for RcAbPhase, RCpAbPhase, and AbSPhase that are closer to those seen in for a normal, synchronous breathing pattern. This reduction in asynchrony may improve symptom management and the quality of life of individuals with dysfunctional breathing. This study provides some interesting insight into the feasibility of using this novel real-time OEP phase angle feedback system for breathing retraining in individuals with a DBP.

## Figures and Tables

**Table 1:** Mean and standard deviation (SD) values for the comparison of breathing parameters between the control group and experimental group at rest, during high intensity exercise, and recovery post-exercise pre- and post- acute breathing retraining intervention.

	Breathing Parameter	Control Group				Experimental Group				Interaction
		Pre		Post		Pre		Post		Effect *p*-Value
		Mean	SD	Mean	SD	Mean	SD	Mean	SD	
Rest	RR (brpm)	15.32	3.22	12.80	4.38	14.63	3.37	11.39	2.55 †	0.728
	RcAbPhase (deg)	–0.34	0.70	–1.39	2.21	−0.05	0.37	−0.08	0.58	0.113
	RCpAbPhase (deg)	−0.33	0.54	−1.14	1.94	−0.02	0.37	−0.01	0.52	0.142
	AbSPhase (deg)	−0.47	0.78	−1.57	2.49	0.04	0.40	−0.18	0.81	0.067
High Intensity Exercise	RR (brpm)	36.41	5.42	26.27	4.97 †	32.90	7.79	25.51	8.27 †	0.398
	RcAbPhase (deg)	−2.97	1.98	−3.65	2.48	−2.89	1.20	−1.39	1.54 †	<0.01 *
	RCpAbPhase (deg)	−1.96	1.46	−2.54	1.93	−2.00	0.93	−0.50	1.31 †	<0.01 *
	AbSPhase (deg)	−2.39	2.34	−3.38	2.61	−2.60	1.35	−0.72	1.97 †	<0.01 *
Recovery	RR (brpm)	23.52	5.35	17.76	2.97 †	23.19	7.51	17.19	4.36 †	0.946
	RcAbPhase (deg)	−0.96	1.15	−1.38	1.95	−1.16	1.21	0.06	1.16 †	<0.05 *
	RCpAbPhase (deg)	−0.56	0.94	−0.86	1.42	−0.73	1.15	0.26	0.86 †	<0.05 *
	AbSPhase (deg)	−0.78	1.26	−1.31	1.52	−1.17	1.61	0.11	1.08 †	<0.01 *

* represents a significant interaction effect between time point (pre- and post-intervention) and group with *p* < 0.05. † represents significant post hoc contrasts between pre- and post-intervention conditions within a given group with *p* < 0.05.

## Data Availability

The data presented in this study are available on request from the corresponding author.
